# Autophagy promotes angiogenesis via AMPK/Akt/mTOR signaling during the recovery of heat-denatured endothelial cells

**DOI:** 10.1038/s41419-018-1194-5

**Published:** 2018-11-19

**Authors:** Pengfei Liang, Bimei Jiang, Yuanbin Li, Zhenguo Liu, Pihong Zhang, Minghua Zhang, Xiaoyuan Huang, Xianzhong Xiao

**Affiliations:** 1 Department of Burns and Plastic Surgery, Xiangya Hospital, Central South University, Changsha, 410008 P. R. China; 2 Department of Pathophysiology, Xiangya School of Medicine, Central South University, Changsha, 410008 P. R. China; 3 Dorothy M. Davis Heart and Lung Research Institute, Division of Cardiovascular Medicine, Department of Internal Medicine, Wexner Medical Center, Ohio State University, Columbus, OH 43210 USA

## Abstract

Our previous study demonstrated that angiogenesis increased during the recovery of heat-denatured endothelial cells. However, the mechanism is still unclear. This study aimed to investigate the relation of autophagy and angiogenesis during the recovery of heat-denatured endothelial cells. A rat deep partial-thickness burn model and heat-denatured human umbilical vein endothelial cells (HUVECs) model (52 °C for 35 s) were used. Autophagy increased significantly in the dermis and HUVECs in a time-dependent manner after heat denaturation and recovery for 2–5 days. Rapamycin-mediated autophagy enhanced the pro-angiogenic effect, evidenced by increased proliferation and migration of HUVECs, and formation of tube-like structures. Autophagy inhibition by 3-Methyladenine (3-MA) abolished the angiogenesis in heat-denatured HUVECs after recovery for 3–5 days. Moreover, heat denaturation augmented the phosphorylation of AMP-activated protein kinase (AMPK) but reduced the phosphorylation of Akt and mTOR in HUVECs. Furthermore, autophagy inhibition by antioxidant NAC, compound C or AMPK siRNA impaired cell proliferation, migration and tube formation heat-denatured HUVECs. At last, the in vivo experiments also showed that inhibition of autophagy by bafilomycin A1 could suppress angiogenesis and recovery of heat-denatured dermis.Taken together, we firstly revealed that autophagy promotes angiogenesis via AMPK/Akt/mTOR signaling during the recovery of heat-denatured endothelial cells and may provide a potential therapeutic target for the recovery of heat-denatured dermis.

## Introduction

Denatured dermis was firstly put forward by Huang et al. in 2001^1^. In deep partial burn wound, denatured dermis appears cell metabolism disorder, function impairment and markedly morphological changes. However, denatured dermis has the ability to restore normal morphology and function after improving the surrounding microenvironment. At present, increasing clinical studies found that preservation of denatured dermis could enhance wound healing and improve the appearance and function of skin^2–4^. However, the underlying molecular mechanism by which denatured dermis could improve wound healing is still unclear.

Angiogenesis, a crucial process for wound healing, is involved in several sequential phases, in which sprout formation is initiated after the degradation of surrounding basement membrane by proteolytic enzymes secreted from endothelial cells, followed by endothelial cell proliferation and migration. Finally, the migrating cells form tube-like structures^5,6^. Our previous studies observed angiogenesis increased during the recovery of heat-denatured HUVECs as evidenced by the increase in endothelial cell proliferation, migration and tube formation^7^. However, the mechanisms of pro-angiogenesis during the recovery of heat-denatured HUVECs have not been explored clearly.

Autophagy is a dynamic process of subcellular degradation. Under starvation, autophagy is critical for cell survival. When autophagy occurs, part of the cellular components is sequestered in autophagosomes and subsequently degraded upon fusion with lysosomes^8^. However, autophagy is not merely a stress response to metabolic perturbations. Autophagy can be induced by a variety of cellular stressors, such as hypoxia, reactive oxygen species (ROS), DNA damage, and aggregation of misfolded proteins^9^.

Increasing evidence has suggested that autophagy is involved in the angiogenic behavior of endothelial cells in vitro^10,11^ and in vivo^12^. However, autophagy seems to have conflicting roles in angiogenesis. In certain circumstances, autophagy induction may also promote endothelial cell death. For example, endostatin, an endogenous angiogenic inhibitor, can induce autophagy and promote human endothelial cells death, and these cells are insensitive to caspase inhibitors but suppressed by the autophagy inhibitor 3-methyladenine^13^. Interestingly, Chen et al^14^. reported that there is a transition from autophagy-mediated cell survival to cell death in hypoxia-treated endothelial cells in a time-dependent manner. However, it remains unknown whether autophagy in endothelial cells influences angiogenesis during the recovery of heat-denatured dermis.

This study aimed to investigate the autophagic activities during the recovery of denatured dermis and HUVECs, the role of autophagy in angiogenesis during the recovery of heat-denatured dermis, and the potential molecular mechanisms. We observed that autophagy was increased significantly during the recovery of heat-denatured dermis and HUVECs. Autophagy was required for pro-angiogenesis during the recovery of heat-denatured dermis, and intracellular ROS could regulate AMPK/Akt/mTOR signaling, enhance autophagy and angiogenesis during the recovery of heat-denatured dermis. Our study provides the first evidence that heat denaturation contributes to pro-angiogenesis in dermis through the production of intracellular ROS and upregulation of autophagic activity.

## Materials and Methods

### Materials

3-MA, rapamycin, Chloroquine, compound C and Bafilomycin A1 were purchased from Sigma Aldrich (St. Louis, MO, USA). DMEM, Opti-MEM, penicillin-streptomycin, antibody against glyceraldehyde−3-phosphate dehydrogenase (GAPDH), AMPK siRNA, and siRNA Transfection Reagent were bought from Santa Cruz Biotechnology (Santa Cruz, CA). Antibodies against LC3, p62 and LAMP2 were bought from Abcam (Cambridge, MA, USA), the antibodies against p-AKT, AKT, AMPK, p-AMPK, mTOR and p-mTOR were from Cell Signaling Technology (Danvers, MA, USA), the recombinant active full-length human Akt1 protein (rAkt1) was purchased from Abcam (Cambridge, MA, USA).

### Animals

Male Sprague-Dawley rats were from the Animal Resource Center of Central South University (Changsha, China), and all animal experiments were performed as per the Guidelines of Animal Experimentation, Medical Ethics Committee of Xiangya Hospital, Central South University (No.201402027).

### Animal model of deep partial-thickness and in vivo experiment

A burn model of deep partial-thickness in Sprague-Dawley rats was performed as described in our previous study^7^. Denatured dermis samples were harvested on days 0, 2, 3, and 5 after deep burn. The back skin of rats were collected for normal control skin samples.In the set of experiments for bafilomycin A1 (Baf 1) and N-acetyl-L-cysteine (NAC), well-established rats were randomly assigned into indicated cohorts. Bafilomycin A1 (0.3 mg/kg, once a day), and NAC (200 mg/kg, once a day) were intraperitoneally injected for 7d. The wound closure percentage was measured at 3d and 7d after surgery by the following formula:Wound closure (%) = [(wound area on day 0—wound area on indicated day)/wound area on day 0] × 100. CD31 expression was detected by immunofluorescent analysis at 0d and 5d after surgery.

### Cell culture and heat denaturation model

HUVECs were obtained from American Type Culture Collection (ATCC, Manassas, VA, USA) and maintained in Dulbecco’s Modified Eagle (DMEM) medium supplemented with 10% fetal bovine serum. The cells were cultured at 37 °C in a humidified incubator with 5% CO_2_. The cells between 3–10 passages were used in the experiments. The heat-denatured cell model was established as previously described^7,14^.

### Cell proliferation assay, Western blot analysis, Transwell, and tube formation assay

Cell proliferation assay, Western blot analysis, Transwell and tube formation assay were performed as described previously^7,15^.

### Immunofluorescent analysis of LC3 puncta and CD31 in heat-denatured dermis

Heat-denatured dermis was fixed with 10% buffered formalin and embedded in paraffin. Immunofluorescence staining of LC3B and CD31 was performed on 4 µm-thickness sections. Images were captured and analyzed under an Olympus IX71 inverted fluorescent microscope (Olympus, ON, Canada). Quantitation of the LC3 puncta was performed by counting 10 high power fields manually for each sample, and the averaged number was calculated.

### Measurement of ROS production

ROS production in HUVECs was evaluated using the oxidant-sensing 2′,7′-dichlorofluorescein diacetate (DCFH-DA) (Invitrogen, Thermo Fisher Scientific, Waltham, MA, USA). After heat denaturation and recovery for 3 and 5 days, the HUVECs were treated with 20 μM DCFH-DA for 20 min at 37 °C. To detect intracellular ROS production, fluorescence was imaged under a Nikon Eclipse TE200 fluorescent microscope. The fluorescence intensity of DCFH was measured using a spectrophotometer (Leica, Heidelberg, Germany) at an excitation wavelength of 480 nm and an emission wavelength of 520 nm.

### AMPK-siRNA transfection

According to the manufacturer’s instructions, the HUVECs (80% confluence) were treated with AMPK or control siRNAs (siRNAs) (human; Santa Cruz Biotechnology) for 48 h. AMPK protein expression in transfected cells was confirmed by Western blot analysis.

### Statistical analysis

All data are presented as mean ± SEM. Student’s *t* test and one-way analysis of variance were used to analyze significant differences using SPSS 16.0 software. A value of *P* < 0.05 represented statistically significant difference.

## Results

### Autophagy increased during the recovery of heat-denatured dermis and HUVECs

**Fig. 1:**
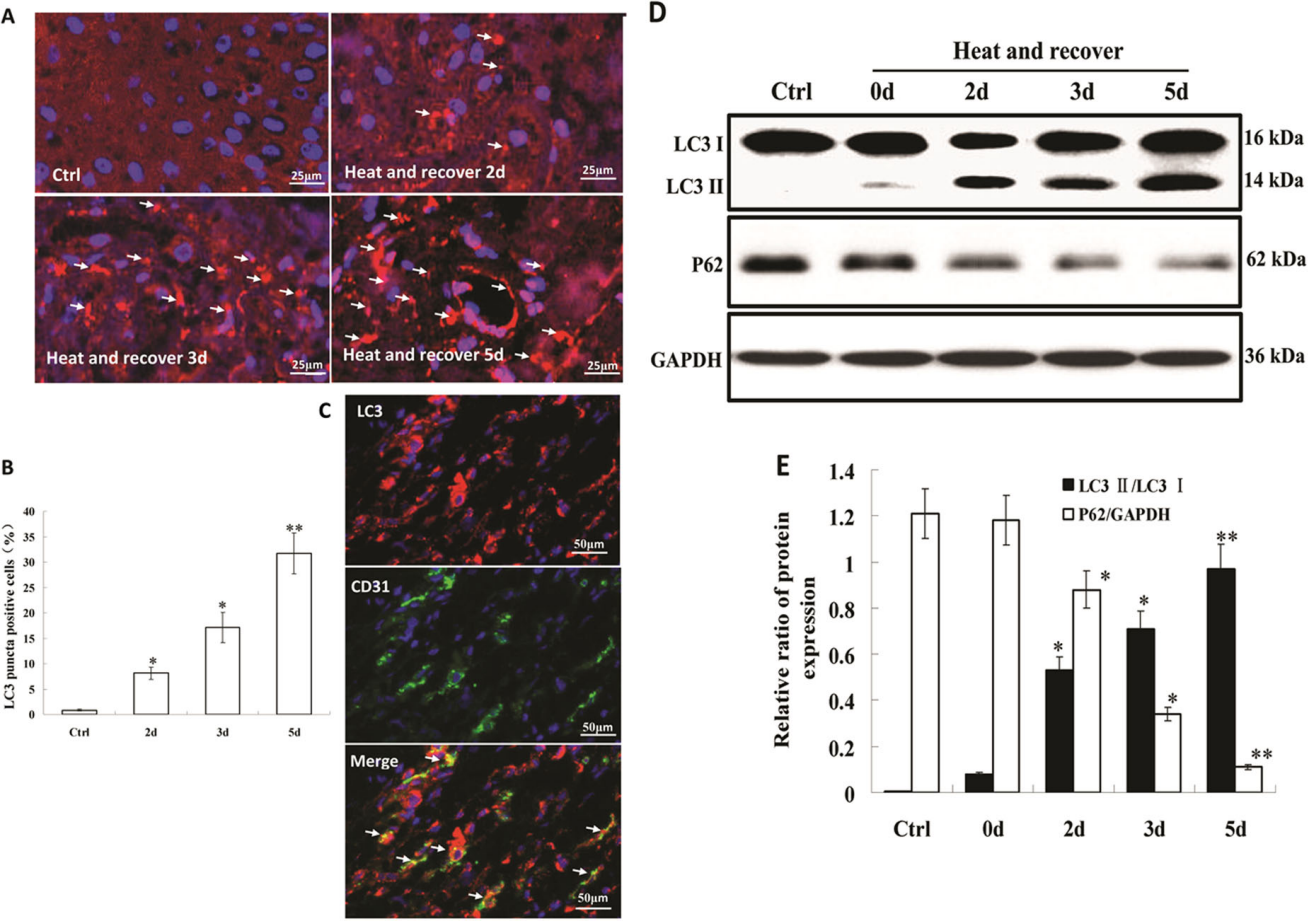
Detection of autophagy during the recovery of heat-denatured dermis. **a** Representative images for the immunofluorescence staining of LC3 (red) and DAPI (blue) during the recovery of heat-denatured dermis (400×). Red dots (white arrows) showed LC3 puncta in dermis. **b** Percentage of LC3 puncta-positive cells in heat-denatured dermis was calculated and analyzed (*P < 0.05 vs. Ctrl group; **P < 0.01 vs. Ctrl group, *n* = 6). The percentage was calculated based on the values in five random fields of each slide under the microscope. **c** Representative images for the immunofluorescence staining of LC3 (red) and CD31 (green) after recovery for 5d of heat-denatured dermis (400×). Arrows indicate the co-localization of LC3 and CD31. **d** Representative blot of LC3 I/II and p62 during the recovery of heat-denatured dermis. **e** The relative ratio of protein expression was calculated and analyzed (*P < 0.05 vs. Ctrl group; **P < 0.01 vs. Ctrl group, *n* = 4)

Immunofluorescent analysis was used to examine the autophagosome formation, as indicated by the redistribution of LC3 from a diffuse distribution to a puncta pattern. As shown in Fig. 1a and b, the formation of numerous LC3 puncta was observed in a time-dependent pattern during the recovery of heat-denatured dermis. More importantly, double-immunostaining of LC3 and CD31 (an endothelial cell marker) showed that LC3 puncta co-localized with CD31 in endothelial cells (Fig. 1c).These results suggested autophagosome formation increased in endithelial cells in denatured dermis. In addition, the levels of phosphatidylethanolamine-conjugated LC3 (LC3 II) and p62 were determined by western-blot. As shown in Fig. 1d, compared to the control group, the ratio of LC3 II/LC3 I showed a remarkable increase in a time-dependent pattern during the recovery of heat-denatured dermis, with the maximum increase (*P* < 0.01) on the fifth day of recovery after burn. However, the expression level of p62 showed a significant decrease in a time-dependent pattern during the recovery of heat-denatured dermis, with the maximum decrease (*P* < 0.01) on the fifth day of recovery after burn.

**Fig. 2:**
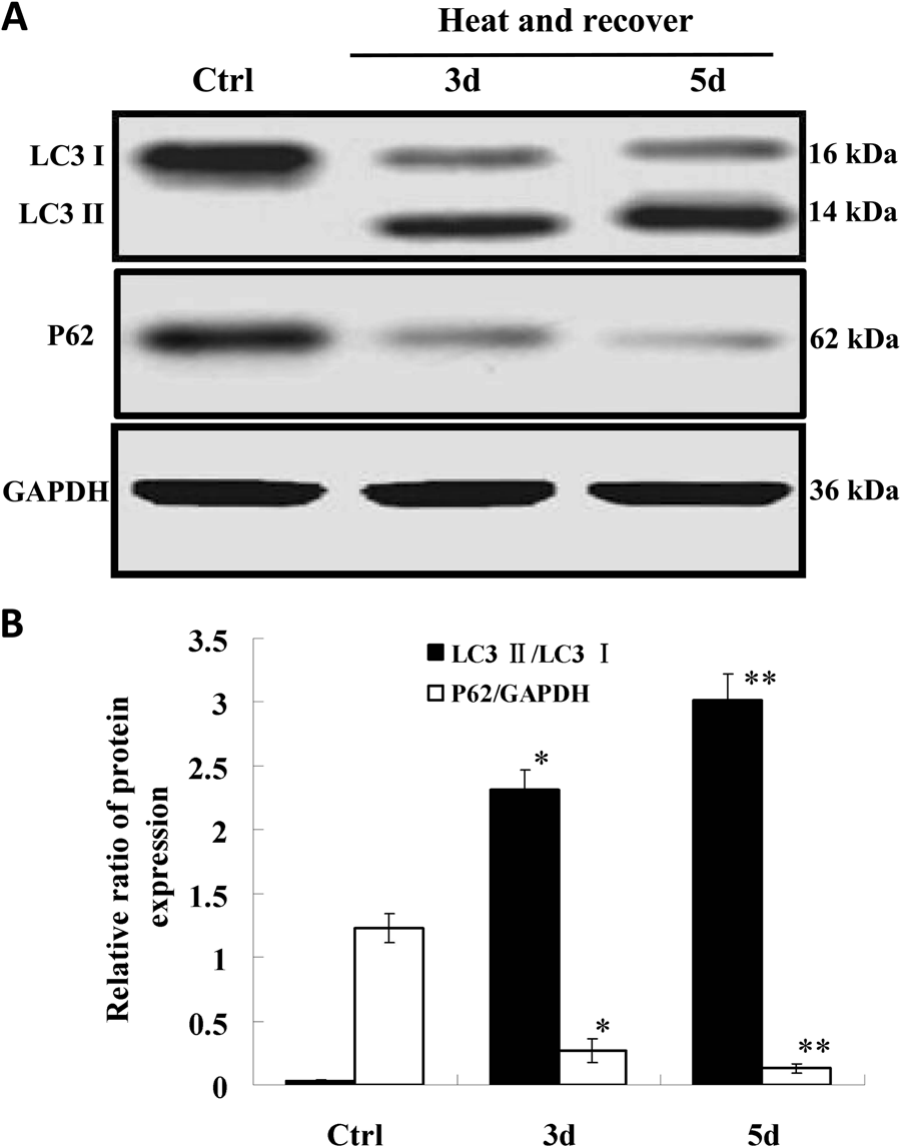
The expression of LC3 II/I and p62 was detected by western-blot during the recovery of heat-denatured HUVEC. **a** Representative blot of LC3 II/I and P62 in heat-denatured HUVEC after recovery for 3 or 5 day. **b** The relative ratio of protein expression was calculated and analyzed (*P < 0.01 *vs*. Ctrl group; **P < 0.01 *vs*. Ctrl group, *n* = 4)

HUVECs were treated with high temperature (52 °C for 35 s) and recovered for 3 and 5 days, and then the levels of LC3 II and p62 expression in cell lysates were examined. As shown in Fig. 2, during the recovery of heat-denatured HUVECs, the ratio of LC3 II/LC3 I was significantly increased after recovery for 3 and 5 days (*P* < 0.01) compared with the controls. While the expression level of p62 was markedly decreased (*P* < 0.01) during the recovery of heat-denatured HUVECs.

### Autophagic flux increased during the recovery of heat-denatured HUVECs

**Fig. 3:**
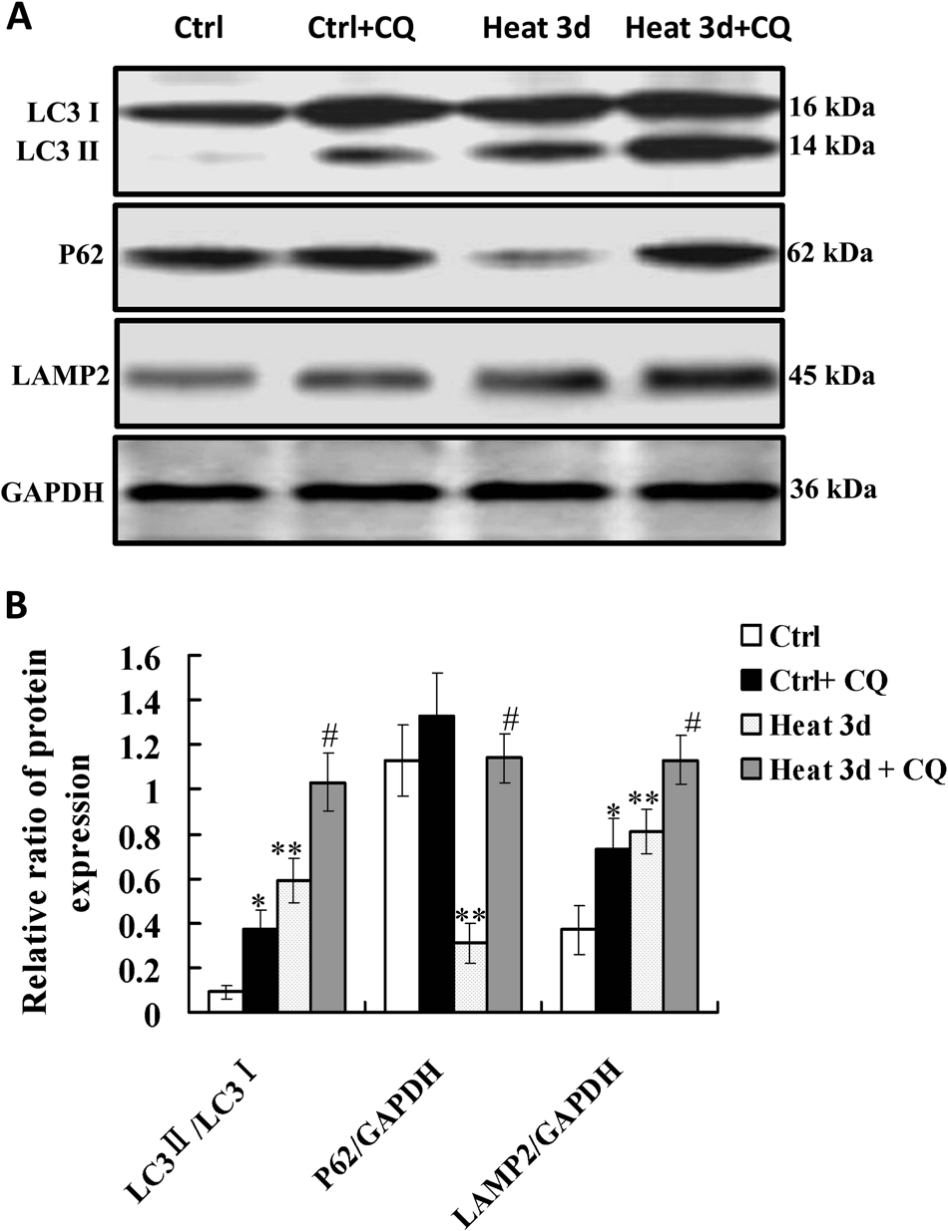
Autophagic flux increased during the recovery of heat-denatured HUVECs. HUVECs were pre-treated with heat denaturation (52 °C, 35 s) and recovered for 3 days, then exposed to chloroquine (CQ) (20 μmol/L) for 4 h. The levels of LC3, p62 and LAMP2 were examined by Western-blot. **a** Representative Western blot image. **b** Densitometry quantification of LC3 II/LC3 I, P62 and LAMP2 normalized to GAPDH expression. The data are the mean ± SEM of three independent experiments. *P *<* 0.05 *vs*. Ctrl group; **P *<* 0.05 *vs*. Ctrl group; ^#^*P* *<* 0.05 *vs*. Heat 3d group

CQ is a lysosomal pH neutralizing agent and can restrain autophagic flux at a late stage. To detect the autophagic flux, we examined the levels of LC3 II, p62 and LAMP2 in the absence or presence of CQ. We found that CQ treatment increased LC3 II, p62 and LAMP2 expression in the cells pretreated with high temperature (52 °C for 35 s) and recovered for 3 days (Fig. 3). These results suggest that cellular autophagic flux was promoted in endothelial cells pre-exposed to high temperature and recovered for 3 days.

### Intracellular ROS production mediates autophagy during the recovery of heat-denatured HUVECs

**Fig. 4:**
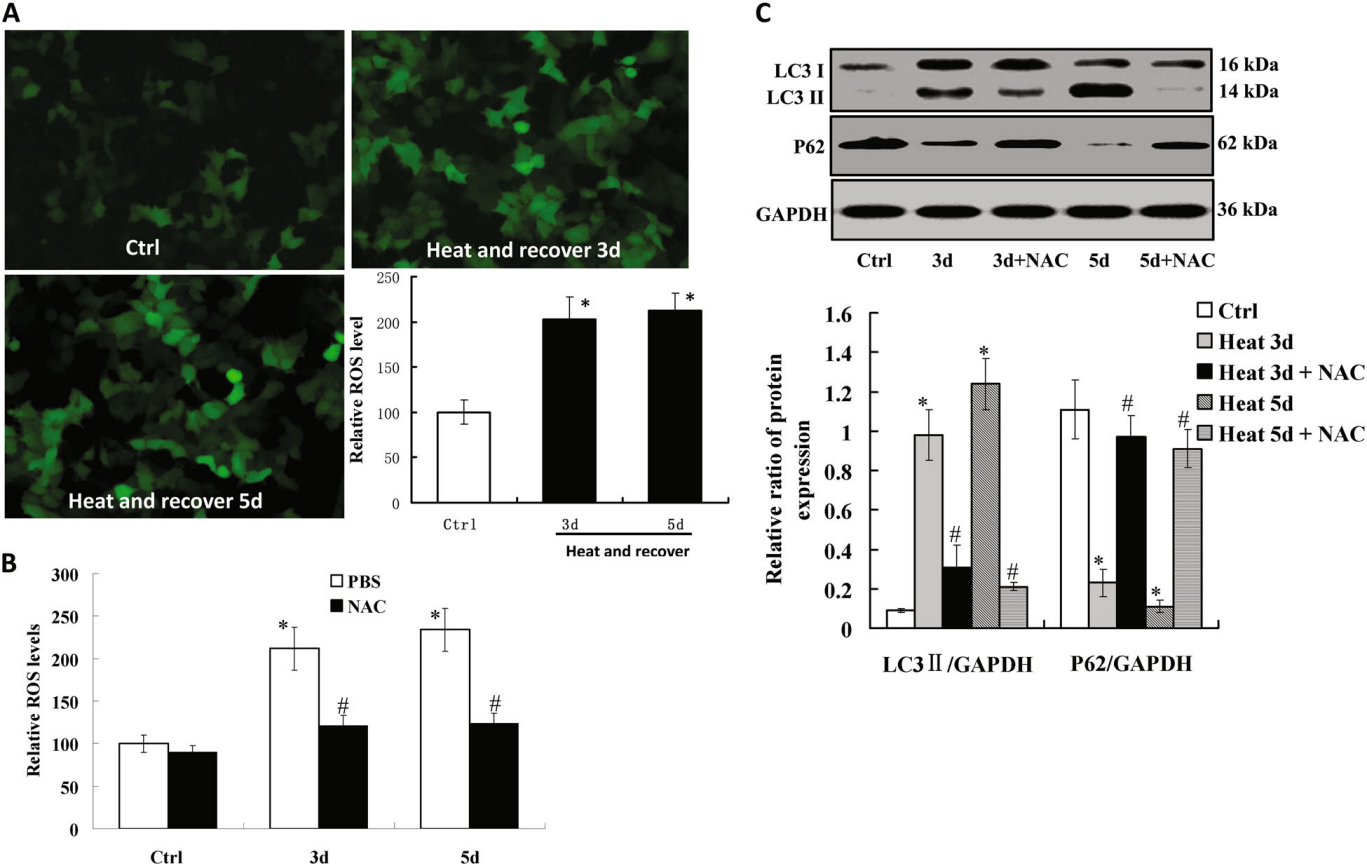
Autophagy is induced by intracellular ROS production during the recovery of heat-denatured HUVECs. **a** The intracellular ROS level was measured by DCFH (green fluorescence). The mean fluorescence intensity was calculated based on the data from at least five different experiments. (*P < 0.01 *vs*. Ctrl group, *n* = 5). B, HUVECs were pre-treated with heat denaturation (52 °C, 35 s) and recovered for 3 or 5 days, and then incubated with 20 μmol/L N-acetylcysteine (NAC). (*P < 0.01 *vs*. Ctrl group, *n* = 5; ^#^*P* < 0.01 *vs*. PBS-treated group, *n* = 5). **c** NAC inhibited autophagy during the recovery of heat-denatured HUVECs. The expression of LC3 and p62 was examined by Western-blot. The relative ratio of protein expression was analyzed. (*P < 0.01 *vs*. Ctrl group, *n* = 5; ^#^*P* < 0.01 *vs*. PBS-treated group, *n* = 5)

Intracellular ROS production is associated with autophagy in HUVECs^16^. Here we examined whether intracellular ROS production mediates autophagy during the recovery of heat-denatured HUVECs. Intracellular ROS production was measured by the fluorescent probe of CM-H2DCFDA. As shown in Fig. 4a, intracellular ROS production was increased in heat-denatured HUVECs compared to the control cells. When heat-denatured HUVECs were incubated with 20 μmol/L of N-acetylcysteine (NAC), a free radical scavenging agent. We found antioxidant NAC significantly suppressed intracellular ROS production (Fig. 4b). Furthermore, the elevated expression of LC3-II in heat-denatured HUVECs was markedly reduced in the presence of NAC, while the reduced expression of p62 in heat-denatured HUVECs was increased in the presence of NAC (Fig. 4c). These results suggest that autophagy in heat-denatured HUVECs is associated with increased oxidative stress.

### Effect of autophagy on cell proliferation during the recovery of heat-denatured HUVECs

**Fig. 5:**
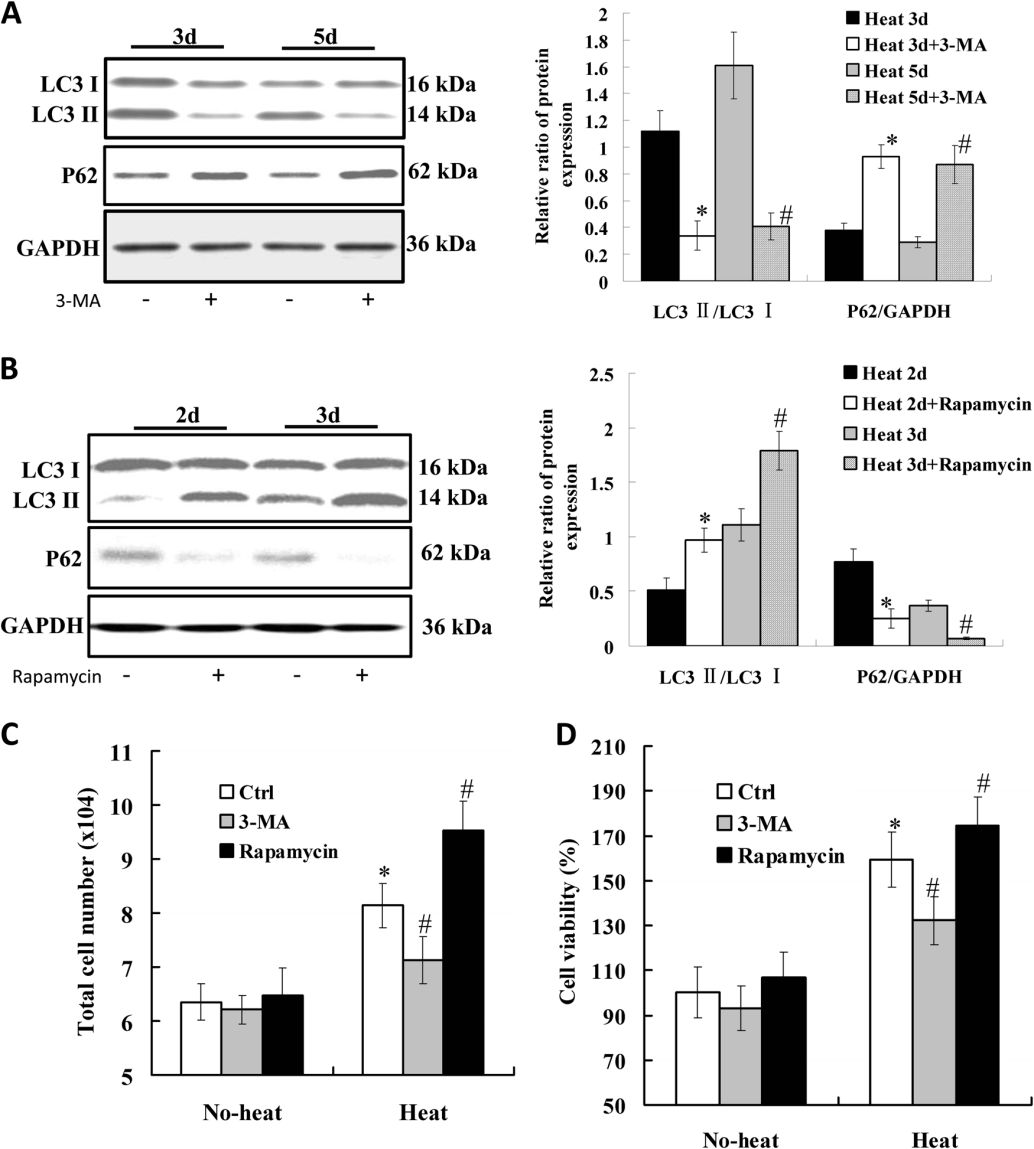
The effect of autophagy on the proliferation during the recovery of heat-denatured HUVECs. **a** The expression of LC3 and p62 in HUVECs pre-treated with heat and recovered for 3 or 5 days, and then incubated with 3-MA (5 mM). The relative ratio of protein expression was analyzed. (*P < 0.01 *vs*. Heat 3d group, *n* = 4; ^#^*P* < 0.01 *vs*. Heat 5d group, *n* = 4). **b** The expression of LC3 and p62 in HUVECs pre-treated with heat denaturation and recovered for 2 or 3 days, and then incubated with rapamycin. The relative ratio of protein expression was analyzed. (*P < 0.01 *vs*. Heat 2d group, *n* = 4; ^#^P < 0.01 *vs*. Heat 3d group, *n* = 4). **c** Cells were trypsinized and counted after pre-treated with heat and recovered for 3 days, and then incubated with 3-MA (5 mM) or rapamycin (200 nM) for 12 h. D, MTT assay was performed. (*P < 0.05 *vs*. Ctrl group, *n* = 6)

To examine the effect of enhanced autophagy on endothelial cell proliferation, HUVECs were pre-exposed to high temperature and recovered for 3 or 5 days, then treated with 5 mM of 3-MA to inhibit autophagy. We observed that 3-MA inhibited LC3-II conversion and p62 degradation during the recovery (3 or 5 days) of heat-denatured HUVECs (Fig. 5a). To further determine whether autophagy is associated with cell proliferation during the recovery of heat-denatured HUVECs, HUVECs were pre-exposed to high temperature and recovered for 2 or 3 days, then treated with rapamycin (200 nM) to induce autophagy. Rapamycin treatment promoted LC3-II conversion and p62 degradation in heat-treated HUVECs (Fig. 5b). We further analyzed the effect of 3-MA and rapamycin on the proliferation of HUVECs after heat treatment and recovery. As shown in Fig. 5c and d, both total cell number counting and MTT assay showed there was significant increase in cell proliferation after heat treatment and recovery for 3 days, and 3-MA treatment significantly inhibited the proliferation of HUVECs after heat treatment and recovery for 5 days, while rapamycin treatment significantly stimulated HUVEC proliferation (*P* < 0.05) after heat treatment and recovery for 3 days.

### Effect of autophagy on the migration of heat-denatured HUVECs

**Fig. 6:**
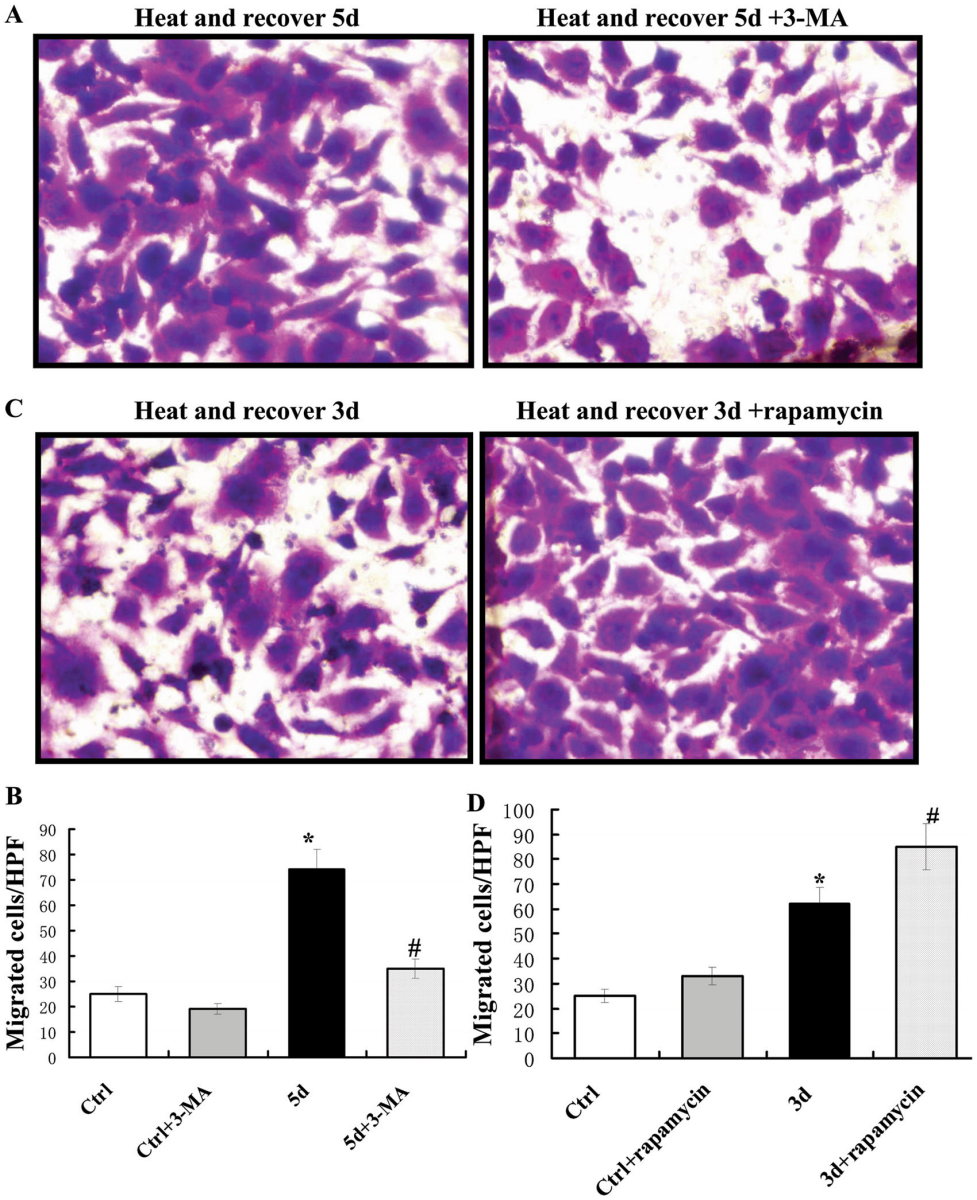
Effect of autophagy on the migration during the recovery of heat-denatured HUVECs. HUVECs pre-exposed to high temperature (52 °C, 35 s) and recovered for 3 or 5 days were treated with 3-MA or rapamycin, and then were used for transwell migration assay. **a** The representative micrographs of migrated cells treated with 3-MA or not after exposure to high temperature (52 °C, 35 s) and recovery for 5 days. **b** The representative micrographs of migrated cells treated with rapamycin or not after exposure to high temperature (52 °C, 35 s) and recovery for 3 days. **c** The number of migrated cells per high-power field (HPF) after 3-MA treatment. (*P < 0.05 *vs*. Ctrl group, ^#^*P* < 0.01 *vs*. Heat and recover for 5d group, *n* = 6). **d** The number of the migrated cells per high-power field (HPF) after rapamycin treatment. (*P < 0.05 *vs*. Ctrl group, ^#^*P* < 0.01 *vs*. Heat and recover for 5d group, *n* = 6)

Our previous study found that endothelial cell migration is increased during the recovery of heat-treated HUVECs^7^. To determine the effect of autophagy on endothelial cell migration, HUVECs were pre-exposed to high temperature and recovered for 5 days, then incubated with 3-MA for 12 h. Cell migration was assessed by the transwell migration assay. As shown in Fig. 6a, 3-MA treatment significantly attenuated the average number of migrated HUVECs per HPF from 73 to 32 (Fig. 6b). To further examine whether increased autophagy promotes HUVEC migration during the recovery of heat-denatured HUVECs, HUVECs were pre-exposed to high temperature and recovered for 3 days, then treated with rapamycin (200 nM) to induce autophagy. As shown in Fig. 6c and d, rapamycin treatment significantly increased the average number of migrated HUVECs per HPF from 62 to 85. These results suggested that autophagy enhanced the migration of heat-denatured HUVECs.

### Effect of autophagy on the formation of tube-like structures of heat-denatured HUVECs

**Fig. 7:**
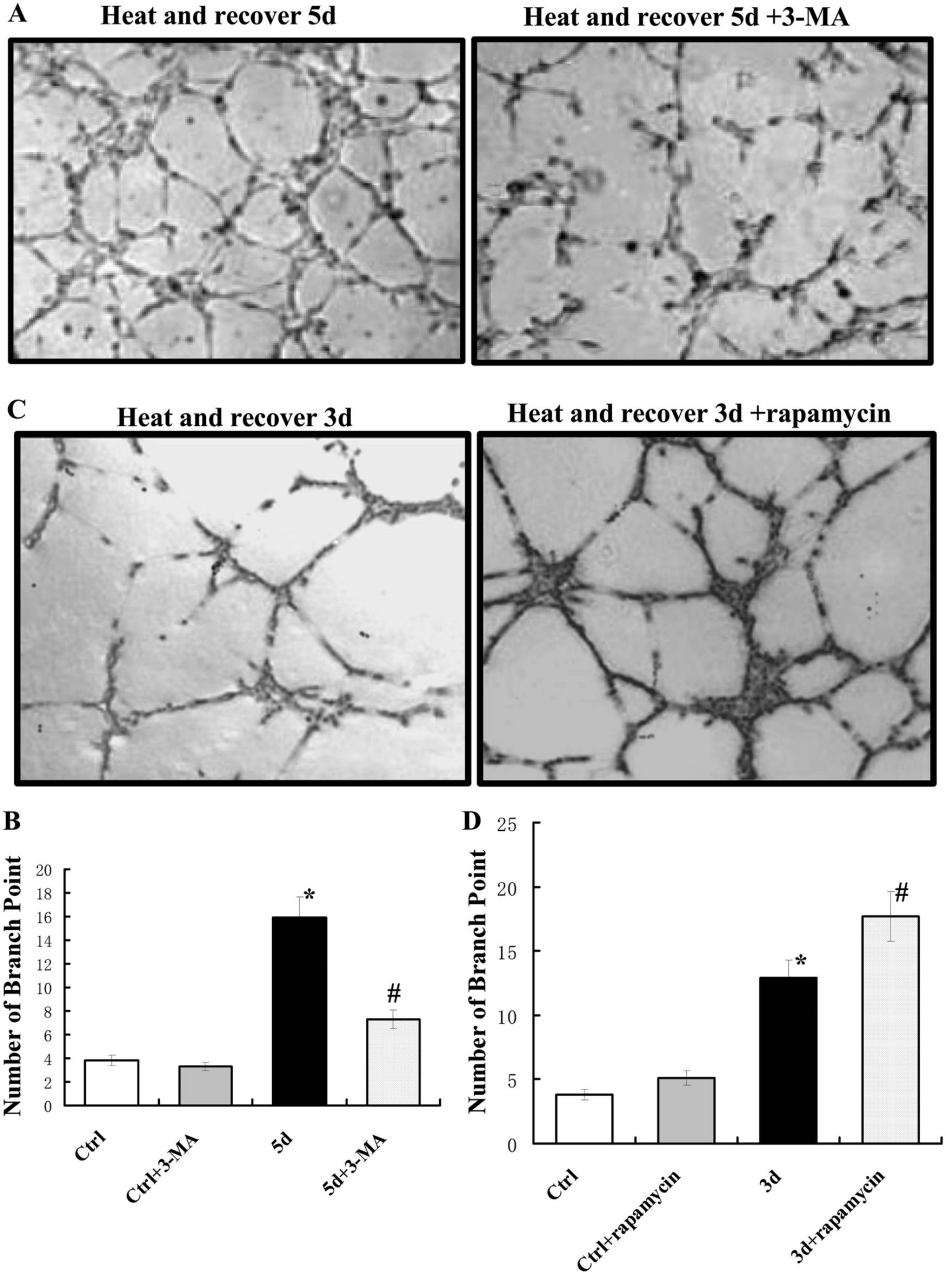
Effect of autophagy on tube formation during the recovery of heat-denatured HUVECs. HUVECs pre-exposed to high temperature (52 °C, 35 s) and recovered for 3 or 5 days (heat) were treated with 3-MA or rapamycin, and then were seeded on Matrigel for tube formation assay. **a** The representative micrographs of tube formation of HUVECs treated with 3-MA or not after exposure to high temperature (52 °C, 35 s) and recovery for 5 days. **b** The representative micrographs of tube formation of cells treated with rapamycin or not after exposure to high temperature (52 °C, 35 s) and recovery for 3 days. **c**, **d** The number of branch point was calculated from 3 independent experiments. (*P < 0.05 *vs*. Ctrl group, ^#^*P* < 0.01 *vs*. Heat and recover 5 d group, *n* = 6). **d** The values of migrated cells treated with rapamycin or not after exposure to high temperature (52 °C, 35 s) and recovery for 3 days (*P < 0.05 *vs*. Ctrl group, ^#^*P* < 0.01 *vs*. Heat and recovery 3d group, *n* = 6)

After heat treatment and recovery for 5 days, HUVECs were incubated with 3-MA for 12 h, and then seeded on basement membrane matrix (Matrigel; BD Bioscience). As shown in Fig. 7a and b, tube-like structures appeared on Matrigel after 24 h of culture, and 3-MA treatment significantly attenuated microvessel tube formation and the number of branching points. On the contrary, treatment with rapamycin significantly increased microvessel tube formation and the number of branching points in HUVECs pre-exposed to high temperature and recovery for 3 days (Fig. 7c, d). Our observations suggest that autophagy increase can effectively increase the ability of HUVECs to form stable tube-like structures in vitro during the recovery of heat-denatured HUVECs.

### The AMPK/Akt/mTOR signaling pathway was required for autophagy and angiogenesis during the recovery of heat-denatured HUVECs

**Fig. 8:**
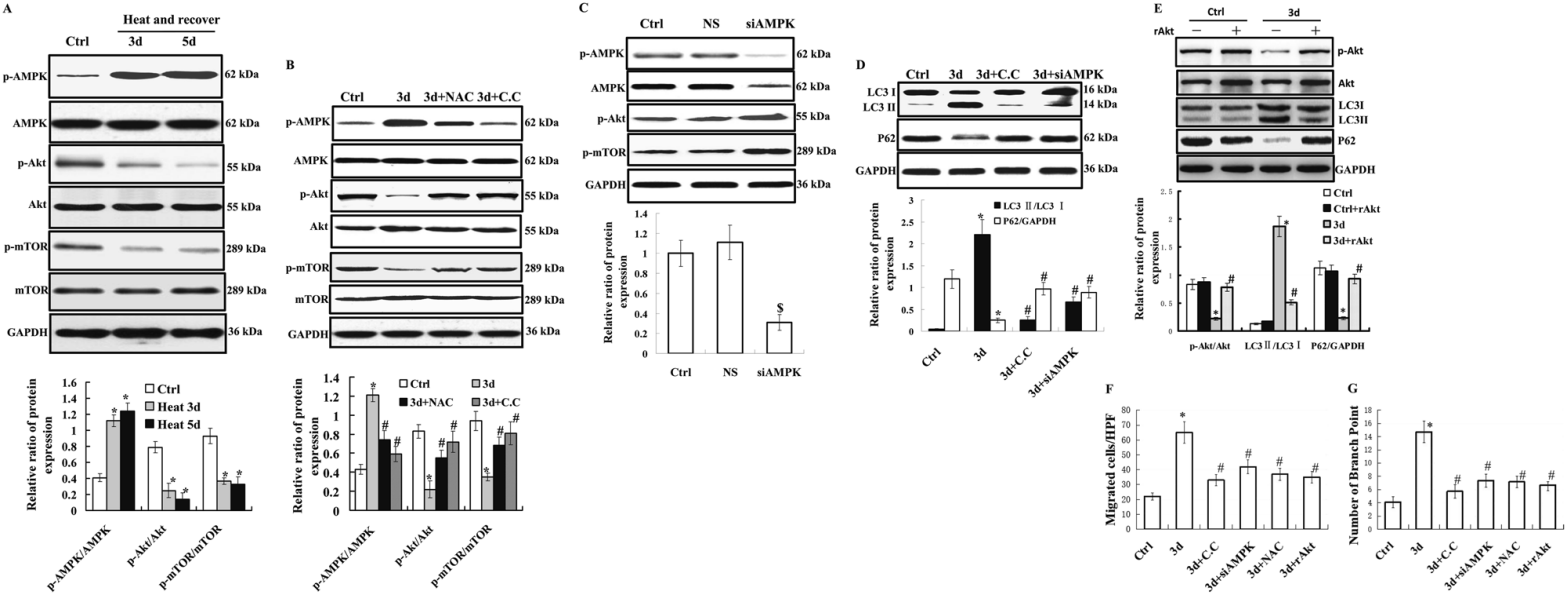
AMPK/Akt/mTOR signaling is required for autophagy and angiogenesis during the recovery of heat-denatured HUVECs. **a** HUVECs were treated with high temperature (52 °C, 35 s) and recovered for 3 or 5 days. A representative Western-blot image is shown (Top), and quantification of protein expression of p-AMPK, p-Akt, and p-mTOR was analyzed (Bottom). **b** HUVECs were pre-treated with high temperature (52 °C, 35 s) and recovered for 3 days (3d) and then treated with NAC or Compound **C**. A representative Western-blot image is shown (Top), and quantification of protein expression of p-AMPK, p-Akt, and p-mTOR was analyzed (Bottom). **c** HUVECs were transfected with AMPK siRNA (siAMPK) or negative siRNA (NS) using Lipofectamine 2000. A representative Western-blot image is shown (Top), and quantification of protein expression of p-AMPK and AMPK was analyzed (Bottom). **d** HUVECs were pre-treated with high temperature (52 °C, 35 s) and then treated with Compoud **C** (C.C) or transfected with AMPK siRNA. The protein expression of LC3 I/II and p62 was examined by Western-blot. A representative Western-blot image is shown (Top), and quantification of protein expression of LC3 and P62 was analyzed (Bottom). **e** HUVECs were pre-treated with heat (52 °C, 35 s) and then treated with rAkt1 (500 ng/ml),the expression of p-Akt, LC3 and p62 was examined by Western-blot. **f** and **g** HUVECs were pre-treated with high temperature (52 °C, 35 s) and then treated with Compoud C (C. C) or transfected with AMPK siRNA. Cell migration (**f**) and tube formation (**g**) of heat-treated HUVECs were analyzed. The data are the mean ± SEM of at least four independent experiments. *P < 0.05, *vs*. Ctrl; ^#^*P* < 0.05 *vs*. heat and recovery 3d; ^$^*P* < 0.05 *vs*. negative siRNA (NS)

It has been reported that AMPK signaling plays an important role in the regulation of cellular autophagy, especially during the autophagosome elongation stage^17–20^. Here we investigated whether AMPK signaling is involved in cellular autophagy during the recovery of heat-denatured HUVECs. Compared with the control group, *p*-AMPK expression was markedly enhanced, while the levels of p-Akt and *p*-mTOR were significantly reduced (Fig. 8a) during the recovery of heat-denatured HUVECs. After heat treatment and recovery for 3 days, HUVECs were then incubated with 20 μmol/L NAC. We observed NAC restored the phosphorylation state of AMPK, Akt and mTOR (Fig. 8b). Moreover, as shown in Fig. 8d, inhibition of AMPK by compound C significantly decreased LC3-I/II expression and increased p62 expression. Transfection of AMPK siRNA reduced AMPK expression by 70% and significantly changed the expression of p-AMPK, p-Akt, p-mTOR and autophagy-associated proteins (*P* < 0.05) (Fig. 8c, d). In addition, recombinant active human Akt1 full length protein (rAkt) was used to activate the Akt pathway, we found that rAkt increased significantly the Akt phosphorylation, and inhibited autophagy in heat-denatured HUVECs. Furtherly, we tested whether NAC, compound C, AMPK siRNA and rAkt influenced the angiogenesis in heat-treated HUVECs. As shown in Fig. 8f, g, NAC, compound C, AMPK siRNA and rAkt treatment significantly decreased the number of migrated cells per HPF and the number of branching points (*P* < 0.05).

### Autophagy is required for angiogenesis and recovery of denatured dermis in rats

**Fig. 9:**
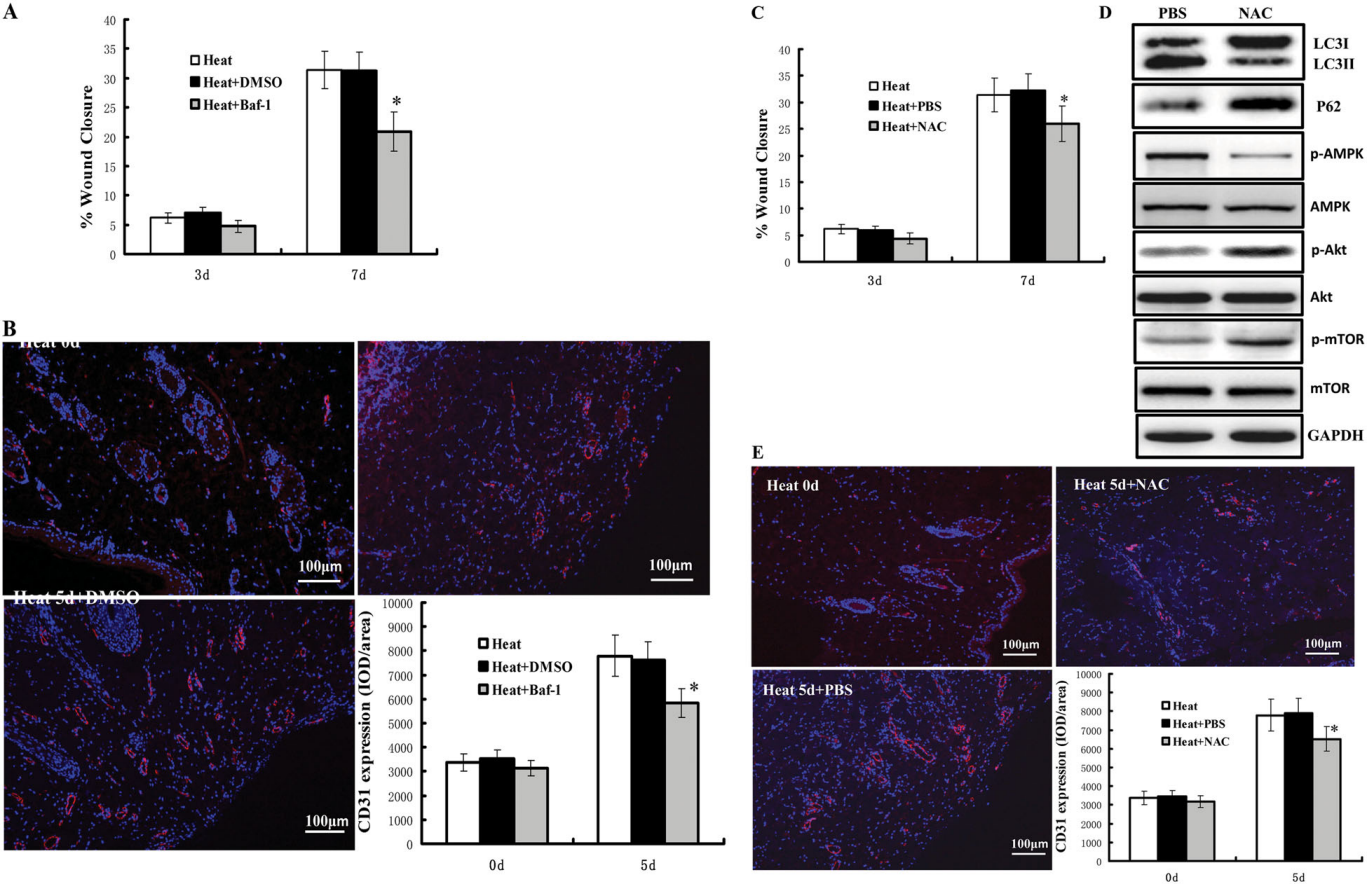
Autophagy is required for angiogenesis and recovery of denatured dermis in vivo. **a** Rate of wound closure in wounds under different treatments is shown. *P < 0.01 *vs*. Heat + DMSO, *n* = 12. **b** Representative images for the immunofluorescence staining of CD31 (red) and DAPI (blue) in heat-denatured dermis at 0d, 5d after heat injury (100×). The integrated optical density (IOD) of CD31 immunostaining was analyzed with Image-Pro® Plus software. *P < 0.01 *vs*. Heat + DMSO, *n* = 5. **c** Rate of wound closure in wounds under different treatments is shown.*P<0.01 *vs*. Heat + PBS, *n* = 12. **d** Western-blot analysis of LC3, P62, AMPK, p-AMPK, Akt, p-Akt, mTOR and p-mTOR in heat-denatured dermis at 5d after heat injury. **e** Representative images for the immunofluorescence staining of CD31 (red) and DAPI (blue) in heat-denatured dermis at 0d, 5d after heat injury (100×). The integrated optical density (IOD) of CD31 immunostaining was analyzed with Image-Pro® Plus software. *P < 0.01 *vs*. Heat + PBS, *n* = 5

Having shown that autophagy is required for the proliferation, migration, and formation of tube-like structures of heat-denatured HUVECs in vitro, we hypothesized that autophagy is essential for angiogenesis during the recovery of heat-denatured dermis. To test this hypothesis, we carried out a study with bafilomycin A1 in rats. As shown in Fig. 9a, the wounds injected intraperitoneally with bafilomycin A1 (Baf 1) showed significantly (*P* < 0.05) lower percentage of wound closure in comparison to the negative control ones, at the 7th day post-injury. Immunostaining with CD31 showed that bafilomycin A1 treatment decreased CD31 expression compared with DMSO treatment (*P* < 0.05) (Fig. 9b). These data indicate that autophagy is required for angiogenesis and wound closure in vivo.

Because intracellular ROS production mediates autophagy during the recovery of heat-denatured HUVECs (Fig. 4), it was important to determine whether these effects could be detected in vivo. As shown in Fig. 9c, at the 7th day post-injury, the wounds treated with NAC showed markedly (*P* < 0.05) lower percentage of wound closure in comparison to the negative control ones. Immunostaining with CD31 also showed that NAC treatment decreased CD31 expression compared with PBS treatment (*P* < 0.05) (Fig. 9e). These data indicate that intracellular ROS production is also required for angiogenesis and recovery of heat-denatured dermis in vivo. Moreover, consistent with the in vitro data, NAC treatment inhibited autophagy in vivo, i.e.,the expression level of LC3II decreased and the expression of P62 increased in heat-denatured dermis. And also, NAC treatment suppressed significantly AMPK phosphorylation, increased Akt and mTOR phosphorylation, indicating that the angiogenesis inhibition mediated by NAC is associated with suppression of autophagy and regulation of AMPK/Akt/mTOR signaling.

## Discussion

Vascular endothelial cells are a kind of cells in the denatured dermis and play important roles in the recovery of heat-denatured dermis. Our previous study found that heat-denatured HUVECs exhibited a strong pro-angiogenic potential during the recovery^7^. In this study, we found that heat denaturation could activate autophagy in HUVECs in vitro and in heat-denatured dermis in vivo. Furthermore, autophagy induced by heat denaturation promoted angiogenesis during the recovery of heat-denatured dermis. First, we demonstrated cellular autophagy was augmented during the recovery of heat-denatured dermis and heat-denatured HUVECs, which is dependent on intracellular ROS production. Second, inhibition of autophagy suppresses cell proliferation, migration and formation of tube-like structure induced by heat treatment (52 °C for 35 s) in vitro. Third, autophagy was required for pro-angiogenesis during the recovery of heat-denatured dermis in vivo; 4) intracellular ROS could regulate AMPK/Akt/mTOR signaling, enhance autophagy and angiogenesis during the recovery of heat-denatured dermis. These data indicate that autophagy is activated, and plays a pro-angiogenic role during the recovery of heat-denatured dermis and its mechanism is probably dependent on production of ROS and regulation of AMPK/Akt/mTOR signaling.

Autophagy is critical for cell survival^21^, and also autophagy is a dynamic flux that includes the initiation, formation, maturation and degradation of autophagosomes^22^. As we all known, the conversion of cytosolic LC3 (LC3-I) to the autophagic vesicle-associated form (LC3-II) through the conjugation of phosphatidylethanolamine is essential for induction of cellular autophagy^23^. p62 is another biomarker to evaluate the autophagic activity. When autophagic flux occurs, p62 is degraded. Thus, Both LC3-II increase and p62 levels decrease is indicatives of autophagic activity^24^. Our results showed that the amount of puncta was increased during the recovery of heat-denatured dermis, LC3 puncta co-localized with CD31 in endothelial cells, and the ratio of LC3-II /LC3-I was increased and p62 was decreased during the recovery of heat-denatured dermis and heat-treated HUVECs. CQ treatment, restraining autophagic flux, resulted in increased LC3 II and p62 expression in the heat-treated HUVECs. These data suggest cellular autophagy flux is augmented during the recovery of heat-denatured dermis and heat-treated HUVECs.

What is the role of cellular autophagy in angiogenesis? Some studies have shown that cellular autophagy may inhibit the angiogenesis in endothelial cells^25^. On the contrary, increasing evidence suggests that cellular autophagy can enhance angiogenesis in endothelial cells^26,27^. Our studies found that cellular autophagy could be induced during the recovery of heat-denatured dermis and heat-denatured HUVECs. Both immunofluorescence analysis and Western-blot results confirmed LC3 II accumulation and p62 decrease and autophagic flux inhibition in the presence of CQ. Moreover, cellular autophagy may promote angiogenesis, as pharmacological inhibition of autophagy by 3-MA significantly impaired cell proliferation, migration and tube formation in HUVECs, while pharmacological induction of autophagy by rapamycin significantly promotes angiogenesis after heat treatment and recovery. Furtherly, bafilomycin A1 (a autophagy inhibitor) was found to inhibit the angiogenesis and recovery of heat-denatured dermis in vivo. Thus cellular autophagy might help wound healing of deep partial burn wound by increasing angiogenesis.

ROS, known as key signaling molecules, are essential for endothelial cell proliferation and migration and can regulate angiogenesis^28^. Recently, ROS are reported to be important in mediating cellular autophagy^29^. In this study, we observed that heat treatment and recovery could significantly induce intracellular ROS production, while inhibition of ROS production by NAC decreased autophagy in HUVECs. Moreover, NAC treatment suppressed significantly AMPK phosphorylation, increased Akt and mTOR phosphorylation, inhibited autophagy and angiogenesis during the recovery of heat-denatured dermis in vivo. These data suggest that the autophagy and angiogenesis during the recovery of heat-denatured dermis are associated with intracellular ROS production.

ROS can induce autophagy through multiple signaling pathways including the AMP-activated protein kinase (AMPK) and mammalian target of rapamycin (mTOR). AMPK has a crucial role in mediating autophagy in endothelial cells in response to metabolic stress or treatments with natural cytoprotective compounds^30,31^. AMPK is a positive regulator of autophagy and suppresses the Akt/mTOR pathway^19,20^. Here we observed up-regulated *p*-AMPK expression and down-regulated *p*-Akt and *p*-mTOR levels during the recovery of heat-denatured HUVECs. These results suggest that AMPK signaling pathway is involved in the regulation of autophagy during the recovery of heat-denatured HUVECs. In addition, our studies found that inhibition of intracellular ROS production by antioxidant NAC decreased autophagy, and ROS influenced the AMPK/Akt/mTOR signaling pathway. To further verify whether the activation of AMPK signaling was related to heat-induced autophagy, AMPK signaling pathway was significantly suppressed by compound C and AMPK siRNA treatment, as evidenced by down-regulated *p*-AMPK level and up-regulated *p*-mTOR level. Moreover, the increase of LC3-II /LC3-I and p62 decrease were dramatically inhibited following compound C and AMPK siRNA treatments. Furtherly, inhibition of AMPK activity, AMPK expression knockdown, and rAkt also inhibited angiogenesis. These results suggest that intracellular ROS production and AMPK/Akt/mTOR signaling pathway may be involved in autophagy and angiogenesis during the recovery of heat denatured dermis.

The Akt/mTOR signaling pathway is a well-known pathway involved in the regulation of autophagy. Akt regulates autophagy mainly through the modulation of mTOR activity, which is an evolutionarily conserved protein kinase. Recent studies suggested that attenuated Akt-mTOR signaling pathway can result in autophagy in Spodoptera litura cultured cell line (SL−1 cell)^32^. Bevacizumab could suppress Akt-mTOR signaling pathway to induce high level of autophagy in glioblastoma cells^33^. Furthermore, Akt/mTOR signal pathway could influence the autophagy and angiogenesis in aortic endothelial cells^10^. Bilobalide B could inhibit autophagy and promote angiogenesis by activating Akt pathway following ischemia/reperfusion injury to the brain^34^.In this study, we demonstrated that Akt-mTOR signaling pathway was inhibited during the recovery of heat-denatured HUVECs (Fig. 8a). Activation the Akt pathway by recombinant active human Akt1 full length protein (rAkt) could inhibit autophagy and then influenced the angiogenesis induced by autophagy in heat-denatured HUVECs (Fig. 8f, g). These results suggest that inhibition of Akt/mTOR pathway increased the autophagy and then promoted the autophagy-induced angiogenesis during the recovery of heat denatured dermis.

Additionally, there are two limitations of our study. The first one is that only HUVECs were used as our model of endothelial cells. Because of different environment, endothelial cells from different tissues probably exhibit some variations in cell growth and morphology^35^. So further studies are required to understand the role of autophagy in angiogenesis and its potential molecular mechanisms. The second limitation is that additional pharmacological tools and siRNAs should be used in the future studies to study their side effects. By the way, 3-MA is a widely used as inhibitor of vesicular protein sorting 34 in autophagy^36^. However, it was reported that 3-MA can also inhibit PI3K activity at high concentrations^37^. To limit these nonspecific effects, we used low concentration (5 μM) in this study.

In conclusion, our study demonstrated that cellular autophagy can promote angiogenesis during the recovery (3 and 5 days) in heat-denatured endothelial cells through increasing intracellular ROS production and activation of AMPK/Akt/mTOR signaling pathway. Inhibition of autophagy by an autophagy inhibitor bafilomycin A1 hindered the angiogenesis and recovery of heat-denatured dermis in vivo. Together, these data uncover new fundamental molecular mechanisms underlying autophagy and therapeutic angiogenesis and provide a novel treatment strategy for deep partial burn wound.

## References

[1] Huang XY, Yang XH, Lei SR (2001). With the preservation of denatured dermis and autoskin grafting to repair of deeply burned hands. Zhonghua Shao Shang Za Zhi.

[2] Yang XH (2005). Long-term result of repair of deeply burned hands with large sheet of split-thickness autoskin grafting with the preservation of denatured dermis. Zhonghua Shao Shang Za Zhi.

[3] Liang P (2012). MicroRNA profiling in denatured dermis of deep burn patients. Burns.

[4] Huang XY (2009). Augmentation of quality of wound healing of deep burn. Zhonghua Shao Shang Za Zhi.

[5] Flegg JA, Byrne HM, Flegg MB, Sean McElwain DL (2012). Wound healing angiogenesis: the clinical implications of a simple mathematical model. J. Theor. Biol..

[6] Hsu YH (2012). Far-Infrared therapy induces the nuclear translocation of PLZF which inhibits VEGF-induced proliferation in human umbilical vein endothelial cells. PLoS. One..

[7] Jiang B (2015). Nucleolin enhances the proliferation and migration of heat-denatured human dermal fibroblasts. Wound Repair Regen..

[8] Glick D, Barth S, Macleod KF (2010). Autophagy: cellular and molecular mechanisms. J. Pathol..

[9] Jiang F (2016). Autophagy in vascular endothelial cells. Clin. Exp. Pharmacol. Physiol..

[10] Du J (2012). Role of autophagy in angiogenesis in aortic endothelial cells. Am. J. Physiol. Cell. Physiol..

[11] Qian HR, Yang Y (2016). Functional role of autophagy in gastric cancer. Oncotarget.

[12] Zhou P, Tan YZ, Wang HJ, Wang GD (2017). Hypoxic preconditioning-induced autophagy enhances survival of engrafted endothelial progenitor cells in ischaemic limb. J. Cell. Mol. Med..

[13] Chau YP, Lin SY, Chen JH, Tai MH (2003). Endostatin induces autophagic cell death in EAhy926 human endothelial cells. Histol. Histopathol..

[14] Liang PF (2013). The expression and proangiogenic effect of nucleolin during the recovery of heat-denatured HUVECs. Biochim. Biophys. Acta.

[15] Jiang B (2014). Nucleolin involved in myocardial ischaemic preconditioning via post-transcriptional control of HSPA1A expression. Cardiovasc. Res..

[16] Li GH (2015). Ox-Lp(a) transiently induces HUVEC autophagy via an ROS-dependent PAPR-1-LKB1-AMPK-mTOR pathway. Atherosclerosis.

[17] Rigacci S (2015). Oleuropein aglycone induces autophagy via the AMPK/mTOR signalling pathway: a mechanistic insight. Oncotarget.

[18] Dai SH (2017). Sirt3 confers protection against neuronal ischemia by inducing autophagy: Involvement of the AMPK-mTOR pathway. Free Rad. Biol. Med..

[19] Chang CH (2017). Resveratrol-induced autophagy and apoptosis in cisplatin-resistant human oral cancer CAR cells: A key role of AMPK and Akt/mTOR signaling. Int. J. Oncol..

[20] Zhong J (2017). Irbesartan ameliorates hyperlipidemia and liver steatosis in type 2 diabetic db/db mice via stimulating PPAR-Υ, AMPK/Akt/mTOR signaling and autophagy. Int. Immunopharmacol..

[21] Kimmelman AC, White E (2017). Autophagy and tumor metabolism. Cell. Metab..

[22] Mizushima N, Komatsu M (2011). Autophagy: renovation of cells and tissues. Cell.

[23] Klionsky DJ (2012). Guidelines for the use and interpretation of assays for monitoring autophagy. Autophagy.

[24] Kim H (2014). The DUSP26 phosphatase activator adenylate kinase 2 regulates FADD phosphorylation and cell growth. Nat. Commun..

[25] Lee SJ, Kim HP, Jin Y, Choi AM, Ryter SW (2011). Beclin 1 deficiency is associated with increased hypoxia-induced angiogenesis. Autophagy.

[26] Xie Y (2011). Protective role of autophagy in AGE-induced early injury of human vascular endothelial cells. Mol. Med. Rep..

[27] Yan J (2010). Autophagy augmented by troglitazone is independent of EGFR transactiva-tion and correlated with AMP-activated protein kinase signaling. Autophagy.

[28] Wong W (2017). New connections: The duality of ROS in angiogenesis. Sci. Signal..

[29] Yan Y, Finkel T (2017). Autophagy as a regulator of cardiovascular redox homeostasis. Free Radic. Biol. Med..

[30] Smith BK (2016). Treatment of nonalcoholic fatty liver disease: role of AMPK. Am. J. Physiol. Endocrinol. Metab..

[31] Wang Q, Liang B, Shirwany NA, Zou MH (2011). 2-Deoxy-d-glucose treatment of endothelial cells induces autophagy by reactive oxygen species-mediated activation of the AMP-activated protein kinase. PLoS. One..

[32] Shao X, Lai D, Zhang L, Xu H (2016). Induction of autophagy and apoptosis via PI3K/AKT/TOR pathways by Azadirachtin A in Spodoptera litura cells. Sci. Rep..

[33] Huang H, Song J, Liu Z, Pan L, Xu G (2018). Autophagy activation promotes bevacizumab resistance in glioblastoma by suppressing Akt/mTOR signaling pathway. Oncol. Lett..

[34] Zheng Y (2018). By activating Akt/eNOS bilobalide B inhibits autophagy and promotes angiogenesis following focalcerebralischemiareperfusion. Cell. Biochem..

[35] Craig LE, Spelman JP, Strandberg JD, Zink MC (1998). Endothelial cells from diverse tissues exhibit differences in growth and morphology. Microvasc. Res..

[36] Mizushima N, Yoshimori T, Levine B (2010). Methods in mammalian au-tophagy research. Cell.

[37] Ito S, Koshikawa N, Mochizuki S, Takenaga K (2007). 3-Methyladenine suppresses cell migration and invasion of HT1080 fibrosarcoma cells through inhibiting phosphoinositide 3-kinases independently of autophagy inhibition. Int. J. Oncol..

